# Symmetries constrain the transition to heterogeneous chaos in balanced networks

**DOI:** 10.1186/1471-2202-16-S1-P229

**Published:** 2015-12-18

**Authors:** Andrea K Barreiro, J Nathan Kutz, Eli Shlizerman

**Affiliations:** 1 Department of Mathematics, Southern Methodist University, Dallas, TX, USA; 2 Department of Applied Mathematics, University of Washington, Seattle, WA, USA

## 

Biological neural circuits display both spontaneous asynchronous activity, and complex, yet ordered activity while actively responding to input. Recently, researchers have demonstrated how this spontaneous behavior underlies computational capabilities in large, recurrently connected networks of firing rate [1,2] and spiking [3] units.

Yet, not all spontaneous activity is equal: complex computations may require the rich phase-space of *heterogeneous chaos*, in which each neuron has a different time-dependent firing rate [2,3]. Here, we address the question of how network connectivity structure may affect the transition to heterogeneous chaos in echo-state networks.

We choose a family of firing-rate networks in which the neurobiological constraint of Dale's Law -- that most neurons are either excitatory or inhibitory -- is satisfied, and in which excitation and inhibition are balanced. We first study the transition to heterogeneous chaos in this setting, using principal component analysis (PCA) to provide a lower-dimensional description of network activity. We find that key characteristics of this transition differ in constrained networks, versus unconstrained networks with similar variability: the transition to heterogeneous chaos occurs at higher coupling strengths, and is variable across specific networks.

These properties are a consequence of the fact that the constrained system may be described as a perturbation from a system with non-trivial symmetries. These symmetries imply the presence of both fixed points and periodic orbits that are an organizing center for solutions, even for large perturbations. In comparison, spectral characteristics of the network coupling matrix [4-6] are relatively uninformative about the behavior of the constrained system.

We next investigated the impact of this structure on the computational capabilities of constrained vs. unconstrained networks. We first examine the response of networks to time-dependent inputs [3]. In comparison to unconstrained networks, constrained networks performed better on population coding of firing rate, but had less ability to separate two different time-dependent inputs in phase space.

In contrast, the delayed transition to chaos had little effect on the ability of constrained networks to reproduce a learning task recently investigated in unconstrained networks [7]. We inspected example networks and found that the addition of the feedback loop quickly moves effective network connectivity away from symmetry and into the chaotic regime at the onset of training.
